# Gastric Duplication Cyst Causing Gastric Outlet Obstruction 

**Published:** 2012-07-01

**Authors:** Muna Al Shehi, Mukhtar Ali, Abdelrahim, Amin El Gohary

**Affiliations:** Paediatric Surgery Department, Mafraq Hospital, Abu Dhabi, United Arab Emirates.

**Keywords:** Gastric duplication, gastric outlet obstruction

## Abstract

This is a case report of a newborn baby with gastric duplication cyst presented with non-bilious vomiting and upper abdominal distension. The diagnosis was suspected clinically and established by ultrasonography and computed tomography. The cyst was completely excised with uneventful recovery.

## INTRODUCTION

Gastrointestinal duplications can occur anywhere in the alimentary tract from the mouth to the anus. Gastric duplication is very rare and accounts for only 4% of gastrointestinal duplications. They are usually cystic and are located on the greater curvature; and have no communication with the gastric lumen. They typically present with partial obstruction. An upper abdominal mass is often palpable on examination. Haematemesis and melaena may rarely occur [1-3]. We are reporting a case of gastric duplication cyst presenting with gastric outlet obstruction.

## CASE REPORT

A 10-day-old male neonate born by normal vaginal delivery at term, presented with recurrent non bilious vomiting. Antenatal ultrasonography revealed a cystic abdominal mass. On examination, there was mild upper abdominal distension with no palpable mass. Laboratory investigations were normal. Plain roentgenogram of abdomen (Fig.1) showed soft tissue mass displacing the stomach upwards. Abdominal ultrasound revealed a cystic mass measuring 42 X 36 mm arising from the greater curvature of the stomach with well defined thick wall displacing the stomach upwards. Computed tomography scan of the abdomen (Fig.2) confirms the ultrasound findings. Gastric duplication cyst was the working clinical diagnosis.

**Figure:**
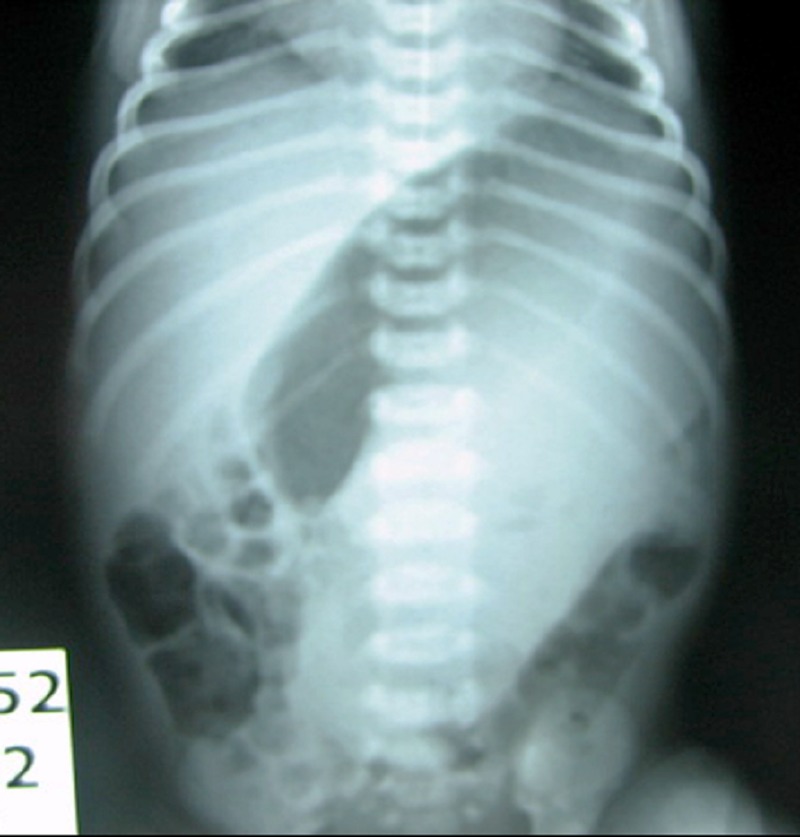
Figure 1: Plain film of abdomen showing gastric displacement by the mass.

**Figure:**
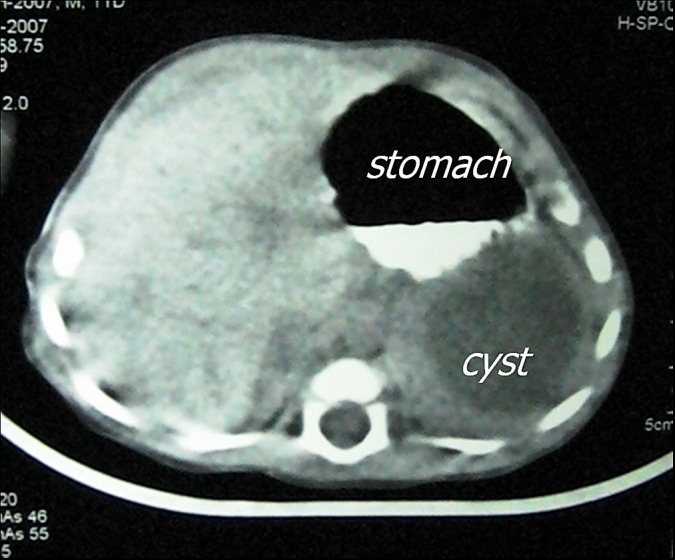
Figure 2: Abdominal CT showing the relation between the cyst and stomach.

Excision of the cyst was initially attempted laparoscopically, but it had to be converted to open laparotomy owing to the difficulty to separate the cyst from the stomach. A large cystic mass with well-defined muscle coat was adherent to the greater curvature of the stomach and not communicating with the gastric lumen or adjacent structures. The cyst was totally excised without entering the gastric lumen. (Fig.3). Gastric seromuscular defect was approximated by interrupted absorbable suture. Histopathologically, the cyst had smooth muscles in the wall and inner lining of gastric mucosa. The child had uneventful post-operative recovery and discharged on the 5th post operative day. 

**Figure:**
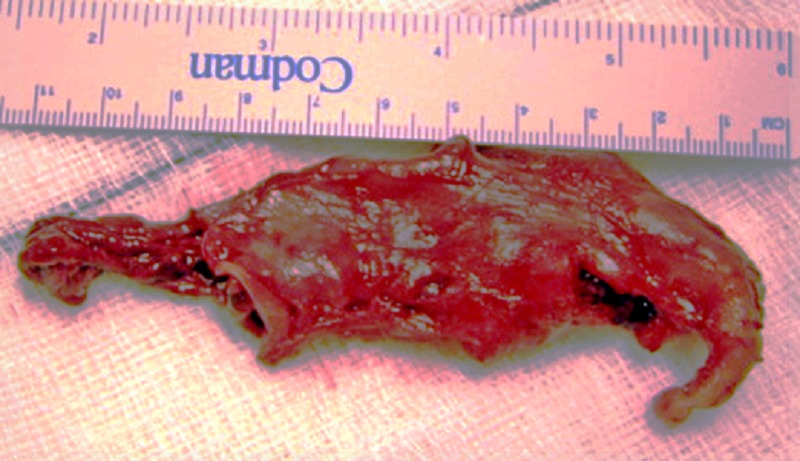
Figure 3: The duplication cyst after excision.

## DISCUSSION

Gastrointestinal (GI) duplication cysts are rare malformations that can occur at any part of the alimentary tract. Although recognized since 1733 by Calder [4], it was not until 1937 that Ladd [5] introduced the term duplication of the intestinal tract to describe a group of congenital anomalies characterized by having a well developed coat of smooth muscle, their epithelial lining is composed of some type of gastrointestinal mucosa and they are attached to the alimentary tract.

Gastric duplications are typically spherical cysts which usually develop on the greater curvature of the stomach. The majority are not communicating with the gastric lumen [2, 6]. The mucosal lining is usually of gastric with ectopic pancreatic tissues in about 40% of cases [6]. Our case contained only gastric mucosa.

They are generally identified within the first year of life [7]. The clinical manifestation is non-bilious vomiting and abdominal mass [2, 3]. Rare clinical presentations are haemat-emesis, chronic anemia, gastrointestinal bleeding; recurrent pancreatitis and perforation with peritonitis and fistulous communication to the spleen or lower lobe of the lung [8-10]. If left untreated, gastric duplications are associated with malignant degeneration [11].

Treatment is complete excision of the cyst. Marsupialization of the cyst wall and stripping of the mucosal lining is the second option if it is not possible to find a plane between the cyst and the stomach. There are few cases in the literature successfully treated by laparoscopic resection [12]. It was not feasible in our case to complete the procedure laparoscopically as the dissection plane was difficult to identify.

## Footnotes

**Source of Support:** Nil

**Conflict of Interest:** None

